# Spray Drying of Chitosan Acid Salts: Process Development, Scaling Up and Physicochemical Material Characterization

**DOI:** 10.3390/md19060329

**Published:** 2021-06-06

**Authors:** Nilia de la Paz, Mirna Fernández, Orestes López, Caridad Garcia, Antonio Nogueira, Leonid Torres, Wilfredo Turiño, Jyrki Heinämäki

**Affiliations:** 1 Center for Drug Research and Development (CIDEM), Ave 26 No.1605, e/ Boyeros and Puentes Grandes, Plaza de la Revolución, Havana City CP 10400, Cuba; nilia.delapaz@cidem.cu (N.d.l.P.); caridad.garcia@cidem.cu (C.G.); antonio.nogueira@cidem.cu (A.N.); 2 Institute of Pharmaceutical and Food Sciences (IFAL), University of Havana, Street 23 # 21425 be/214 and 222, La Lisa, Havana City CP 13600, Cuba; mirnafc@ifal.uh.cu; 3 Faculty of Science and Food Engineering, Technical University of Ambato, Ave de Los Chasquis y Rio Payamino, Ambato 180207, Ecuador; od.lopez@uta.edu.ec; 4 Biomolecular Chemistry Center, Street 200 Corner 21, Playa, Havana City CP 11300, Cuba; leonidtorresmaro@gmail.com; 5 Cuban Center for Advanced Studies, Valle Grande, La Lisa, Havana City CP 17100, Cuba; turinolazaro733@gmail.com; 6 Institute of Pharmacy, Faculty of Medicine, University of Tartu, 1 Nooruse St, 50411 Tartu, Estonia

**Keywords:** chitosan salt, pharmaceutical excipient, spray drying, organic acid, process parameters, physical material properties, scale up

## Abstract

We investigated a spray drying process for preparing water-soluble salts of high molecular weight chitosan (CH) intended for pharmaceutical excipient applications. CH was derived from chitin of marine lobster origin (*Panulirus argus*). The effects of organic acid (acetic or lactic acid) and the ratio (difference) of inlet/outlet air temperature (140/90 °C or 160/100 °C) on spray drying were studied. The yield of spray-dried CH salt powders ranged from 50% to 99% in laboratory and industrial-scale processes. The spray-dried dry powder of CH salts consisted of spherical agglomerated particles with an average diameter of 36.2 ± 7.0 µm (CH acetate) and 108.6 ± 11.5 µm (CH lactate). After dispersing the spray-dried CH salt powder samples in purified water, the mean particle sizes obtained for the CH acetate salts were 31.4 nm (batch A001), 33.0 nm (A002) and 44.2 nm (A003), and for the CH lactate salts 100.8 nm (batch L001), 103.2 nm (L002) and 121.8 nm (L003). The optimum process conditions for spray drying were found: an inlet air temperature of 160 ± 5 °C, an outlet temperature of 100 ± 5 °C and an atomizer disk rotational speed of 18,200 min^−1^. The X-ray powder diffraction (XRPD) and differential scanning calorimetry (DSC) results confirmed the amorphous state of the CH salts. The ^1^H nuclear magnetic resonance (NMR) and Fourier transform infrared (FT-IR) spectra of CH acetate and lactate salts verified that the spray drying process does not affect the polymer backbone. In conclusion, both laboratory and industrial-scale spray drying methods for preparing water-soluble acid salts of CH are reproducible, and the physicochemical properties of the corresponding CH acid salts are uniform.

## 1. Introduction

Chitosan (CH) is a cationic polyamine and a partially deacetylated derivative of chitin, which is the second most abundant polymer in nature and a supporting material of crustaceans, insects and fungal mycelia. For commercial applications, chitin is isolated from the shells of marine crustaceans, shrimps and crabs [1]. CH is sparingly soluble in water, but it is soluble in dilute aqueous solutions of most organic acids. CH is capable of salt formation, and the acetate, ascorbate, lactate and malate salts of CH are water soluble. Today, CH is of major importance in the pharmaceutical and food industry due to its excellent properties, such as biocompatibility, biodegradability, non-toxicity, absorption and antimicrobial characteristics [2,3,4,5]. The potential applications of CH as a novel pharmaceutical excipient have been highlighted in several reports [5,6,7,8,9,10,11,12]. In addition, the regulatory authorities have approved the use of CH, and a monograph relating to CH hydrochloride was included in the fourth edition of the European Pharmacopeia (2002) [13] and in the Handbook of Pharmaceutical Excipients [14].

Spray drying is a low-cost continuous manufacturing process that is widely used in the pharmaceutical and food industry for the modification of powder particle and solid-state properties, granulation and microencapsulation (liquids or solids). For pharmaceutical applications, spray drying is commonly applied in the production of direct compression tableting excipients and amorphous solids and in the encapsulation of fragrances, oils and flavours [15,16]. In addition, spray drying is the method of choice for the production of thermally-sensitive active pharmaceutical ingredients (APIs). More recently, spray drying is increasingly being applied in the pharmaceutical formulation of large biomolecules and biologicals [17]. The spherical shape and uniform particle size of the spray-dried particles promote powder flow, capsule filling and tablet compression characteristics [16].

The process conditions and the solvent system applied in spray drying have a significant influence on the physical properties of the final powder [17]. Water is the preferred solvent for most pharmaceutical wet processes since the use of organic solvents produces toxicity and environmental problems [16]. Spray drying is a rapid and reproducible method with good scale-up potential [17,18,19]. However, the dependence of many process variables in spray drying may become a challenge in terms of reproducibility and ability to scale up the process [20]. On the other hand, spray drying is a flexible process offering substantial variation in the encapsulation matrix and is adaptable to commonly used processing equipment [21,22]. Furthermore, spray drying can be well adapted on an industrial scale, which is a true advantage over other related fabrication methods which are only applicable on a laboratory scale [23].

Spray drying has been successfully applied in the preparation of CH suspensions, salts and several types of microspheres and matrices for controlled release applications [24,25,26,27,28,29,30,31,32,33]. To date, virtually all spray-dried CH salts are based on chitin of marine shrimp or crab shell origin. In our previous study, we demonstrated that CH derived from marine lobster (*P. argus*) chitin can be salified with acetic, lactic and citric acids by means of spray drying [34]. The particles of CH salts were spherical in shape, and the particle characteristics were dependent on the process temperature used in spray drying. We also found that the CH acetate salt form had a higher moisture content compared with CH lactate and citrate salts. CH citrate and acetate salts with a higher exothermic temperature were found to be more stable than CH lactate salts [34].

The aim of this study was to investigate the spray drying of the water-soluble acetate and lactate salts of high molecular weight CH intended for pharmaceutical excipient applications. The CH used in the spray drying experiments was derived from chitin of lobster (*P. argus*) origin. The effects of organic acid (acetic or lactic acid) and the ratio (difference) of inlet/outlet air temperatures (140/90 °C or 160/100 °C) on spray drying were studied. The physicochemical properties of CH salts, such as particle size, shape and surface morphology, bulk powder properties, physical solid state, thermal behaviour and chemical purity, were investigated by means of scanning electron microscopy (SEM), X-ray powder diffraction (XRPD), differential scanning calorimetry (DSC), liquid ^1^H nuclear magnetic resonance (NMR) spectroscopy and Fourier transform infrared (FT-IR) spectroscopy.

## 2. Results

### 2.1. Yield

The yield of spray-dried CH salt powders ranged from 50% to 99% in laboratory and industrial-scale processes. More specifically, the yield of spray-dried CH salt powders (acetate and lactate) on a laboratory scale was 50% (or higher), but as the process was scaled up to an industrial scale, the yields of spray-dried CH acetate and lactate powders were 99% and 98%, respectively. The statistical analysis (Table 1) showed that the organic acid used in spray drying affected the yield of spray-dried CH salts, and this effect was statistically significant (*p* < 0.05). It is obvious that some material is lost due to the limited fly of particles in a drying air stream inside a spray-dryer chamber, and thus not reaching a collector unit and cyclone separator. The use of lower molecular mass (MM) acetic acid as a solvent increased the yield of a spray-dried CH salt powder. Increasing the ratio (difference) of inlet/outlet air temperature (DT) in the spray drying process resulted in an increased yield of CH salt powders with both solvents. However, the effect of DT on the yield of CH salts in spray drying was not statistically significant (Table 1).

### 2.2. Particle and Powder Properties

**Figure 1:**
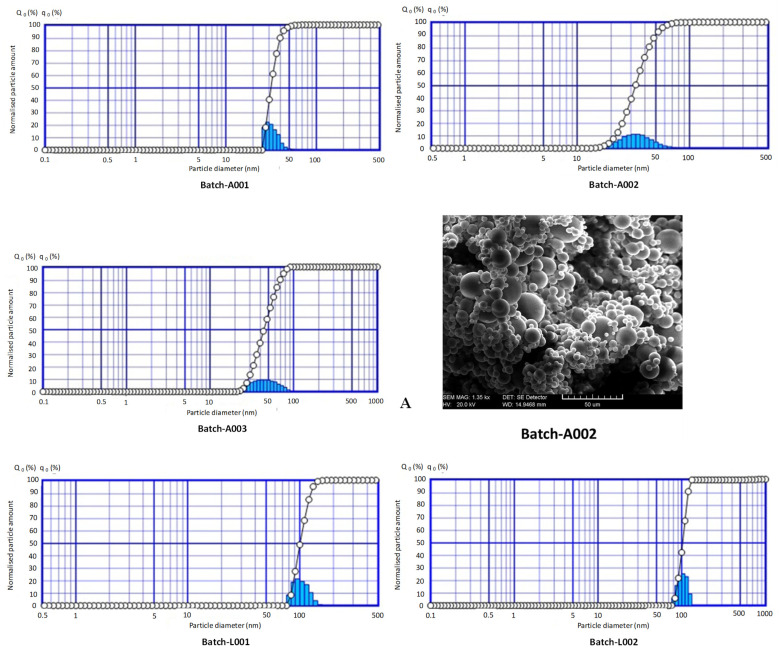

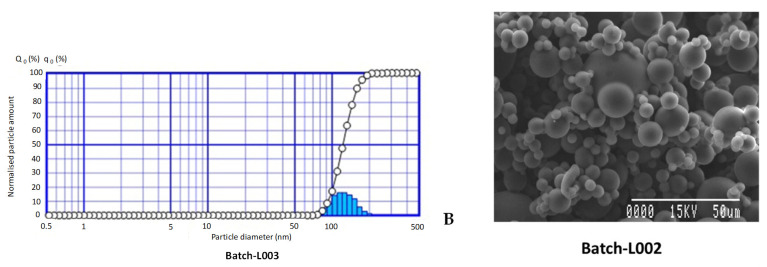
Particle size and size distribution of chitosan (CH) acid salts (based on the volume occupied by the particles), and the representative scanning electron photomicrographs (SEMs) on the CH acetate salt particles (batch-A002, **A**) and CH lactate salt particles (batch-L002, **B**). Scale bar is 50 μm. The CH salts were spray dried at an inlet/outlet air temperature of 160/100 °C in an industrial-scale process (three parallel batches).

The particle size and size distribution of spray-dried CH acid salts (based on the volume occupied by the particles) are shown in Figure 1. The CH acetate and lactate salts were spray dried at inlet/outlet air temperatures of 160/100 °C in the industrial-scale process (three parallel batches). The spray-dried dry powder of CH salts consisted of spherical agglomerated particles with an average diameter of 36.2 ± 7.0 µm (CH acetate) and 108.6 ± 11.5 µm (CH lactate). After dispersing the spray-dried CH salt powder samples in purified water, a colloidal dispersion was formed by the polymer. The mean particle sizes obtained for the CH acetate salts were 31.4 nm (batch A001), 33.0 nm (A002) and 44.2 nm (A003), and for the CH lactate salts 100.8 nm (batch L001), 103.2 nm (L002) and 121.8 nm (L003). The higher particle size of CH lactate salts could be explained by the higher water activity of CH lactate salts compared to the water activity of CH acetate salts, and because of the increase in the humidification of the sample [34]. As seen in Figure 1, CH acid salts also presented a unimodal particle size distribution, suggesting the homogeneity of the particle size in the colloidal dispersions of each salt. This is a characteristic outcome for the spray-dried pharmaceutical excipients [14]. In addition, the spray-dried CH salt particles exhibited hollow spheres with an exceptionally smooth surface (Figure 1).

The bulk density, tapped density, Hausner ratio and Carr index of the spray-dried CH acetate and lactate salts are summarized in Table 2. The tapped densities of the spray-dried CH acetate powder and spray-dried CH lactate powder were within 0.460–0.470 g/cm^3^ and 0.460–0.500 g/cm^3^, respectively. The bulk density values for the CH acetate and lactate powders were 0.230–0.260 g/cm^3^ and 0.260–0.280 g/cm^3^, respectively. The tapped and bulk density differences of the spray-dried CH salts, however, were not statistically significant (*p* > 0.05).

### 2.3. Solid-state and Thermal Properties

**Figure 2:**
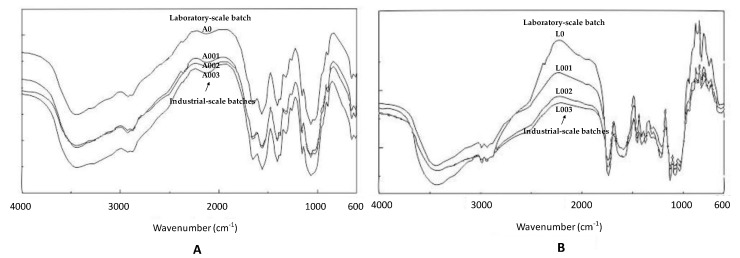
Fourier transform infrared (FT-IR) spectra of chitosan (CH) salt powders. The first curve from the top in both (**A**) and (**B**) represents the FT-IR spectrum of spray-dried CH salt obtained in a laboratory-scale process. (**A**) CH acetate: the first industrial-scale batch A001 (the second curve from the top), second batch A002 (the third curve) and third batch A003 (the fourth curve). (**B**) CH lactate: the first industrial-scale batch L001 (the second curve from the top), second batch L002 (the third curve) and third batch L003 (the fourth curve). The CH salts were spray dried at an inlet/outlet air temperature of 160/100 °C in a laboratory and industrial-scale process.

The FT-IR spectra of spray-dried CH acid salts are shown in Figure 2 (industrial-scale CH acetate batches A001, A002, A003 and CH lactate batches L001, L002, L003 and the corresponding laboratory-scale batches).

The FT-IR spectra exhibited broad bands in the range of 3500–3400 cm^−1^ (which is assigned to OH stretching), thus indicating intermolecular hydrogen bonding. The intense peaks at 1550–1600 cm^−1^ and the weak peaks near 1400 cm^−1^ (attributed to carboxylate anion stretching) were observed in the FT-IR spectra of CH acetate salts. Moreover, the FT-IR spectra of spray-dried CH acetate and lactate salts show a distinct peak (-NH_2_) at 1582 cm^−1^ (Figure 2).

**Figure 3:**
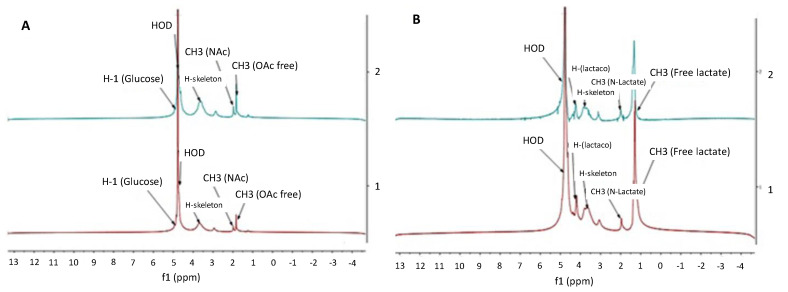
^1^H-NMR spectra of chitosan (CH) salt powders. (**A**) The upper (blue) curve represents the ^1^H-NMR spectrum of spray-dried CH acetate salt obtained in a laboratory-scale process, and the lower (red) curve is the ^1^H-NMR spectrum of spray-dried CH acetate salt produced in an industrial-scale process (batch A002). (**B**) The upper (blue) curve represents the ^1^H-NMR spectrum of spray-dried CH lactate salt obtained in a laboratory-scale process, and the lower (red) curve is the ^1^H-NMR spectrum of spray-dried CH lactate salt produced in an industrial-scale process (batch L002). The CH salts were spray dried at an inlet/outlet air temperature of 160/100 °C in a laboratory and industrial-scale process.

The representative ^1^H-NMR spectra of CH acetate (batch A002) and lactate (batch L002) salts are shown in Figure 3. The CH acid salts were spray dried in both laboratory and industrial-scale processes (three parallel batches). As seen in Figure 3, the spray-dried CH salts show a signal at 3 ppm (H_2_). The proton bands of H_3_, H_4_ and H_6_ were separated from the respective HOD bands by drawing a smooth curve on the low magnetic field side. The terms H_3_, H_4_ and H_6_ refer to the ring positions of aminoglucose.

**Figure 4:**
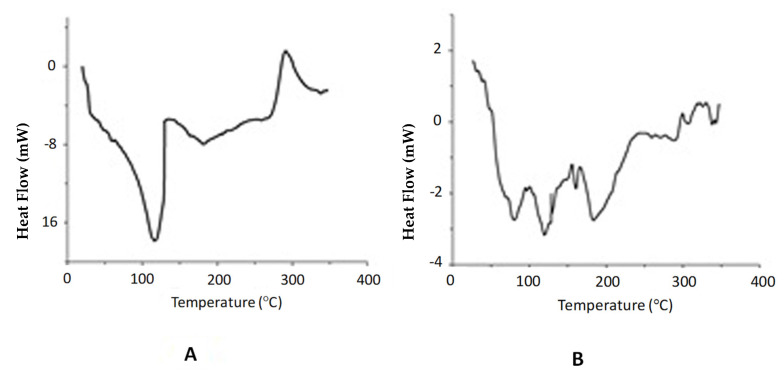
Differential scanning calorimetry (DSC) thermograms of chitosan (CH) salt powders. (**A**) CH acetate batch A002 and (**B**) CH lactate batch L002. The CH salts were spray dried at an inlet/outlet air temperature of 160/100 °C in an industrial-scale process.

Figure 4 shows the representative DSC thermograms of CH acetate and lactate salts (CH acetate batch A002 and CH lactate batch L002 were spray dried in an industrial-scale process). The closely related DSC thermograms were obtained with the corresponding CH salts obtained in the other parallel batches (data not shown). As seen in Figure 4, the DSC thermograms of all samples are characterized by broad endothermic peaks in the temperature range of 70–120 °C. In addition, the CH acetate salt showed another endothermic peak in the temperature range of 150–200 °C. Both endothermic peaks are related to the weight loss of the CH acid salt samples [35].

**Figure 5:**
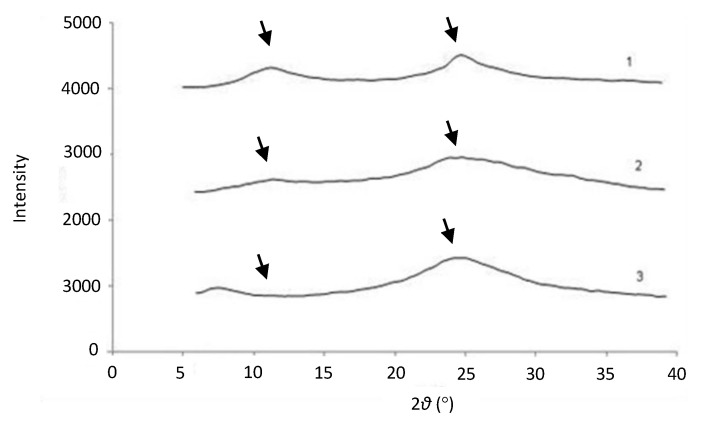
X-ray powder diffraction (XRPD) patterns of chitosan (CH) and CH salt powders. (1) CH, (2) CH acetate batch A002 and (3) CH lactate batch L002. The CH salts were spray dried at an inlet/outlet air temperature of 160/100 °C in an industrial-scale process.

Figure 5 shows the XRPD pattern of CH and the corresponding patterns of CH acetate and CH lactate salts obtained in the industrial-scale spray drying process. The spray-dried CH acid salts exhibited an amorphous solid-state structure. CH and both spray-dried CH acid salts displayed a wide XRPD peak at around 20–25° (2θ), which is predominant to CH derived from chitin of crustacean origin.

### 2.4. Chemical Purity

A summary of the chemical purity results of spray-dried CH acid salts is shown in Table 3. The values for the ash content (0.3–0.5%), matter insoluble in water (0.3–0.4%), heavy metals (<0.5 ppm) and loss on drying (2.5–4.8%) showed the good chemical purity of the spray-dried salts of CH. Slightly higher values for the loss on drying were obtained with CH acetate compared with the respective values for CH lactate. The degree of deacetylation (molar) of CH acetate was also slightly higher than that of CH lactate.

## 3. Discussion

Spray drying offers numerous advantages in preparing water-soluble CH salts. Spray-dried powders of CH salts can be obtained by dissolving CH in an acidic aqueous solution, such as acetic, glutamic, lactic or hydrochloric acid. In the spray drying process, a polymer solution is nebulized, inducing the fast evaporation of the solvent. The temperature used in the spray drying process depends on the boiling point of the solvent. The polymer and API are exposed to this temperature only for a very short time since the atomized solution (droplets) quickly cools down under solvent evaporation. The critical material and process factors affecting the final spray-dried particle, powder properties and particle size include feed solution concentration, viscosity of the solution, boiling point of the solvent, nozzle system, feed rate, inlet/outlet air temperature and atomization/drying gas affect [17].

In our study, CH acetate and lactate salt powders were prepared using spray drying on a laboratory and industrial scale. The effects of the two spray drying parameters (type of organic acid and ratio of inlet/outlet air temperature) on the yield of CH salt powders were investigated (Table 1). The yield of spray-dried CH salt powders was found to be dependent on the organic acid used as a solvent system. Acetic acid has a lower molecular mass (acetic acid 60.06 and lactic acid 90.08) and a lower ebullition point (acetic acid 118.2 °C and lactic acid 122 °C); thus, it is a more volatile acid compared with lactic acid [36]. Therefore, the yield of a spray-dried CH acetate powder was slightly higher than that of a spray-dried CH lactate powder. The production yield values also corresponded well with the viscosity of CH acidic solutions used in spray drying (273.6 mPas for CH acetate and 249.0 mPas for CH lactate). All aqueous CH solutions used in this study were readily atomized in a spray drying process.

Spray drying is a typical solvent evaporation process and the solvent (in the form of droplets) is removed very quickly due to heat energy [17]. Since the boiling point of water is 100 °C, the process inlet air temperature used in spray drying needs to be higher than this value for successful spray drying. The selection of the relatively high inlet temperatures (140 °C and 160 °C) for a spray drying process was based on our earlier study on the spray drying of CH acidic salts [34], and the fact that in a spray drying process an immediate evaporation of the polymeric droplets enhances the formation of spherical powder particles. In a spray drying process, when the solute to be dried is dissolved or dispersed in a non-organic solvent such as water, the inlet temperature of higher than 100 °C is required to enable a complete evaporation of water. If the inlet temperature is adjusted at approximately 100 °C, the output temperature will be in the range of 40 °C and 60 °C, which would not enable the evaporation of all water (resulting in a completely wet final product). Furthermore, the use of such high inlet and outlet temperatures does not cause the degradation of the final product, since by atomization the solids are exposed to high temperatures only for a few seconds. We found that the yield of spray-dried CH salt powders appeared to increase slightly as the ratio of inlet to outlet air temperature was increased. However, due to the limited number of parallel spray drying experiments, this effect was not able to be fully verified. It has been shown previously with aqueous CH solutions that if the inlet air temperature in spray drying is set to below 140 °C, the solvent in the droplets cannot fully evaporate [21,30,37].

Regarding process performance, we found that the application of inlet/outlet air temperature of 160/100 °C resulted in the higher yield of spray-dried CH salt powders compared with the set of inlet/outlet air temperature of 140/90 °C. Spray-dried CH salt particles did not adhere to the drying chamber walls and the outlet orifices of the disk atomizer were not blocked, thus indicating that the inlet/outlet air temperature used (160/100 °C) was close to optimal. A spray drying setup equipped with a disk atomizer was also found to be suitable for processing CH salts. Disk atomizers are versatile and can even be used for spray drying higher viscosity fluids. These atomizers have hammers to facilitate the recovery of dry powder, and therefore the yield of a spray-dried product is higher [38].

The applicability and performance of materials intended for pharmaceutical excipient uses are very much dependent on the particle properties (i.e., size, shape and surface morphology) and bulk powder properties (flowability). In our study, the particle shape and surface morphology of the spray-dried CH salts greatly differed from that of non-spray-dried CH (which consisted of mainly irregular particles; reference is made to [34]). The spray-dried CH acetate and CH lactate powders consisted of spherical agglomerated particles with a mean diameter of 36.2 ± 7.0 μm and 108.6 ± 11.5 μm, respectively. The spray-dried CH salt particles exhibited hollow aggregated spheres with an exceptionally smooth surface. In addition, the particle size of the spray-dried CH lactate acid salt powder was significantly larger than that of the corresponding CH acetate salt powder. This can be explained by the fact that the solid content of the CH lactate solution (97 g/L) used in spray drying was higher than the concentration of CH acetate solution (46 g/L), thus resulting in the larger particle size of the final product. These findings are also in accordance with the results reported in literature [34,39].

The bulk powder properties of the spray-dried CH lactate and acetate salts are summarized in Table 3. Since the particles of the spray-dried CH salts were hollow spheres, this suggests a presumably lower particle true density compared with that of the non-spray-dried material (CH). It is well known that spray drying produces solids in an amorphous state which do not have an ordered structure or a defined shape. With respect to the packing characteristics of the powders, it would be expected that the spray-dried small spherical particles are prone to readily packing together, thus leading to a lower ratio of volume to mass and consequently to a higher tapped density. The tapped and bulk density of the present spray-dried CH salts were very closed to each other, and the difference was not statistically significant (*p* > 0.05). It is generally known that spray-dried acid salts exhibit poor powder flow properties, and the values of the Carr index and Hausner ratio for the CH salts also indicated that both CH salts possess very poor flow characteristics (Table 3). The resting angle of the CH salts was not determined since the spray-dried powders did not flow through a measuring tube. In summary, the physical properties of the powder of the two spray-dried CH salts prepared in the industrial-scale spray dryer coincided with those of CH acetate salts reported in literature [35,40].

To verify the effects of spray drying on the chemical structure, the FT-IR and ^1^H-NMR spectroscopy analyses were performed for the spray-dried CH acid salts obtained in both the industrial-scale and the corresponding laboratory-scale processes (Figure 2 and Figure 3). The broad FT-IR spectroscopy bands in the range of 3500–3400 cm^−1^ (which is assigned to OH stretching) suggest intermolecular hydrogen bonding. The NH stretching could also overlap in the same region of the FT-IR spectra. With all batches of the spray-dried CH salts, these bands at 1597 and 1615 cm^−1^ are diminished, suggesting that the –NH groups are protonated. The carboxylate band of –COO^−^ at 1556 cm^−1^ appeared in the FT-IR spectra of all CH acid salts.

The FT-IR spectra for spray-dried CH acetate salts exhibit intense peaks at 1550–1600 cm^−1^ and weak peaks near 1400 cm^−1^ (attributed to carboxylate anion stretching). The spray-dried CH lactate, in turn, shows a large peak (-NH_2_) at 1582 cm^−1^ (Figure 2). The large shift of this vibration to higher wavenumbers compared with the typical wavenumbers of amino groups suggests the formation of a carboxylate between the COO^−^ groups of the acids and the NH_3_^+^ groups of CH [41]. Consequently, it is reasonable to assume that there is an ionic interaction between CH and acids.

We observed that the spray-dried CH salts show a signal at 3 ppm (H_2_) in a ^1^H-NMR spectroscopy spectra, and a small resonance line (near 4.6 ppm) is to be assigned to the H_1_ band due to acetamidoglucose residue (Figure 3). The terms H_3_, H_4_ and H_6_ refer to the ring positions of aminoglucose. The present results suggest that N-acetyl glucosamine units have survived the spray drying process. As seen in Figure 3, the proton signals corresponding to H_6_, H_4_, H_3_, H_6_ and H_5_ are retained, thus suggesting that the spray drying process and its scaling up do not affect the CH polymer backbone.

As shown in Figure 3, the CH acetate salt shows characteristic signals at approximately 1.8 ppm due to the free CH_3_. With the CH lactate salt, the corresponding signal can be observed at 1.5 ppm. In the case of CH lactate, the higher signal intensity is attributed to the presence of the free acid, unlike CH acetate which exhibits a weaker signal. In the vicinity of 2 ppm, the resonance band due to the CH_3_ residue of N-acetyl CH_3_(NAc) can be observed (Figure 3). For both CH salts, the signal appears as a singlet at 2 ppm, thus indicating the potential interaction between the corresponding acid and the free amine. Two resonance lines appear at around 2.1 ppm. The upfield and downfield resonances were assigned to the CH_3_ of N-acetyl residue and to the acetic acid produced by hydrolysis, respectively. The acid hydrolysis at 70 °C, which results in the increase in CH_3_COOH, was reported by Hirai et al. [42]. Nunthanid et al. [40] reported the conversion of the CH acetate molecular structure to N-acetylglucosamine at higher temperatures. This suggests that both CH acetate and lactate salts are formed as a result of spray drying.

Our results suggest that N-acetyl glucosamine units have remained (“survived”) in a spray drying process. Commercial CHs usually have a minimum deacetylation rate of 60%. It is well-known that the thermo-alkaline deacetylation procedures of chitin enable deacetylated products to be obtained by 75–85%. Thus, the procedure used in our current study (and in our previous works) enables to obtain the corresponding salts and the units of glucosamine are detected. The monomeric form of chitin was detected in the polymer chain of both CH salts, suggesting an incomplete deacetylation of the chitin, which relates to the signals shown in the NMR spectra. Based on the signals analyzed in the NMR spectra and our previous results of ^13^C-NMR [34], it was shown that the CH acid salts were formed as a result of a spray drying process. In the case of CH acetate salt, the CP-MAS ^13^C spectra showed an additional resonance at 180 ppm assigned to a carbonyl group, thus indicating the presence of an acetamide functional group. The conversion of CH acetate to an acetamide form depends on a spray drying process. Nunthanid et al. (2004) reported that the conversion of the molecular structure of CH acetate to N-acetylglucosamine is most likely occurred that at high temperatures [35].

We investigated the physical solid-state properties (DSC, XRPD) of the CH acetate and CH lactate salts obtained in the industrial-scale spray drying process (Figure 4 and Figure 5). The spray-dried CH acid salts show an amorphous solid-state structure. This can be explained by the rapid evaporation of the aqueous medium in spray drying, which produces amorphous spherical particles that have a low glass transition temperature. These results are in good agreement with those obtained by Fernández Cervera et al. [34]. With the spray-dried CH acid salts, no XRPD peak is observed at 9–10° (2θ), thus indicating the absence of a hydrated polymorph of CH [43]. A wide XRPD peak of CH at around 20–25° (2θ) is characteristic for CH derived from chitin of crustacean origin [44]. In our study, the DSC thermograms obtained with the spray-dried CH acetate and lactate salts were in good agreement with the XRPD results.

It is evident that the particle and physicochemical solid-state properties of CH acid salts obtained in laboratory-scale spray drying are slightly different from those of the CH acid salts obtained in the industrial-scale spray drying process. This is due to the differences in the dimensions of the spray dryers and the droplet size of a spray, which can directly affect the wall contact of particles and the rate of evaporation, respectively. Therefore, the results obtained in the laboratory-scale spray drying process can be considered only indicative, and the process development needs to be completed with a spray dryer in an industrial-scale production set-up. This has also been pointed out in literature [17,39,45]. In this work, we found some challenges in designing and preparing CH acetate and lactate salts using a laboratory-scale spray dryer (Mini Spray Dryer Büchi B-191, Switzerland) and directly transferring the corresponding formulations to an industrial-scale process (San Young, Korea).

The chemical purity analysis performed on the CH salts showed the good chemical purity of the final spray-dried material (Table 3). The pharmaceutical quality attributes of the spray-dried CH salts were in line with the pharmacopoeian (Ph.Eur.) [13] quality requirements. Chitosan and its salts are inert, biocompatible and biodegradable materials, characteristics that are important for the materials intended for pharmaceutical excipient applications. It is evident that the spray-dried CH salts investigated in our study could find pharmaceutical excipient uses, e.g., in the formulation of oral solid dosage forms (e.g., tablets and capsules), controlled drug release applications and wound healing therapy.

## 4. Materials and Methods

### 4.1. Materials

Chitosan (CH) was obtained from N-deacetylating lobster (*P. argus*) chitin in accordance with the procedure reported in our previous studies [46,47]. The molecular weight and the degree of deacetylation of CH were 309 kg/mol and 83%, respectively. Lactic acid (BDH, London, UK), acetic acid (Merck, Darmstadt, Germany) and all other reagents used were of analytical grade.

### 4.2. Spray Drying of Chitosan Salts

The effects of the molecular mass (MM) of organic acid (X_1_) and the ratio (difference) of inlet/outlet temperature, DT (X_2_), on the particle size, shape, surface morphology and water content were studied using a simple full factorial experimental design. The total number of experiments was eight (Table 4). Statgraphics Plus software (version. 5.1, StatPoint Technologies, Inc., Warrenton, VA, USA) and Statgraphics Centurion XV (version. 15.2.05, StatPoint Technologies, Inc., Warrenton, VA, USA) were used to carry out statistical variance analysis.

The CH dispersions containing 4.0 g of the polymer were prepared using lactic acid 10% (*w/w*) or acetic acid 10% (*w/w*) as an aqueous solvent system. The concentration of solids was 97.0 g/L and 46.0 g/L, respectively. The dispersions were stirred for 24 h at room temperature until a homogeneous appearance (without any particles) was achieved. Then, the CH dispersions were filtered and subsequently spray dried. The acidic solutions of CH were spray dried using a laboratory-scale spray dryer (Mini Spray Dryer Büchi B-191, Büchi Labortechnik AG, Flawil, Switzerland), with the settings of the inlet/outlet temperature set at 140/90 °C and 160/100 °C, respectively (Table 4) [34]. For process optimization, the yield of each spray drying experiment was determined (=a major response).

### 4.3. Scaling Up of Spray Drying

The industrial-scale spray drying of CH salts was carried out with a San Young (Seoul, Korea) spray dryer. The diameter of the drying chamber was 2.50 m and the total height 1.5 m. The inlet air temperature was kept at 160 ± 5 °C and the outlet air temperature at 100 ± 5 °C. The rotational speed of the atomizer disk was 12,736 min^−1^. The spray-dried powders were subsequently dried for 3 h. Three batches of both CH acetate and lactate salts were produced in order to investigate the effects of scale-up on spray drying and final product properties. An industrial-scale batch size was 50 L.

### 4.4. Characterization of Chitosan Salts

#### 4.4.1. Viscosity of Aqueous Chitosan Salt Solutions

The viscosity of aqueous CH salt solutions was measured as 20.0 ± 0.1 °C by a viscometer (HAAKE RV-20, Karlsruhe, Germany) at 0–500 1/s prior to spray drying. The viscosities of the CH acetate and lactate solutions were 273.6 mPas and 249.0 mPas, respectively.

#### 4.4.2. Particle Size, Shape and Morphology

The particle size, shape and surface morphology of CH acid salts were studied by scanning electron microscopy, SEM (FEG-MEV JEOL 7500F, Jeol GmbH, Freising, Germany). The SEM was operated at an acceleration voltage of 2 kV. The samples were coated with a carbon layer with a thickness of estimated 15 nm (not measured) and imaged at different magnifications. The particle size of spray-dried CH powders was determined by means of laser diffraction combined with dielectrophoresis (Shimadzu IG-1000, Kyoto, Japan). The particle size measurements were conducted by a particle-in-liquid (PIL) method. For PIL particle size measurements, 300 μL of 1% (*w/v*) aqueous solution (0.25 mg/25 mL) was used as a medium. The frequency used was set at 350 kHz and the voltage at 30 V. The measurement time was 100 ms.

#### 4.4.3. Water Content

The moisture content of CH salts was determined in triplicate using the Karl Fischer method (Mettler DL35, Mettler-Toledo GmbH, Schwerzenbach, Switzerland).

#### 4.4.4. Fourier Transform Infrared (FT-IR) Spectroscopy

The FT-IR spectra of the samples were collected with an FT-IR spectrometer (Vertex 70/Bruker, Ettlingen, Germany). The IR specimens were mounted as KBr discs. A total of 64 cumulative scans were taken in transmission mode with a resolution of 4 cm^−1^ and in the frequency range of 4000 to 400 cm^−1^.

#### 4.4.5. Differential Scanning Calorimetry (DSC)

Differential scanning calorimetry (DSC) thermograms of CH and CH salt powders were obtained using a differential scanning calorimeter (DSC Q100 TA, New Castle, DE, USA). Samples were accurately weighed out in aluminium pans and sealed. In this method, a small hole was created in the top of the pan in order to allow the release of moisture. A nitrogen purge with a flow rate of 50 mL/min was used in the furnace. The heating rate was 5 °C/min and the temperature scanning range was from 0 to 300 °C.

#### 4.4.6. Liquid ^1^H-NMR Spectroscopy

High resolution liquid ^1^H-NMR spectroscopy was carried out with a Bruker Advance DPX 250 FT NMR spectrometer (Bruker Corp., Billerica, MA, USA) using D_2_O as a solvent at a concentration of 60 mg/mL. The solutions were freeze dried three times to exchange labile protons. The spectra were recorded at 250 MHz, 13 MHz and a temperature of 25 °C. The 90° pulse width was at 9 µs. The spectral width and data points were 3000 Hz and 32 K points, respectively, and ^1^H chemical shifts were expressed in ppm downfield from the signal for tetramethylsilane as an external reference.

#### 4.4.7. X-ray Powder Diffraction (XRPD)

X-ray powder diffraction (XRPD) patterns were obtained using a variable temperature X-ray diffractometer (D8 Advance Bruker AXS GMBH, Karlsruhe, Germany) (VT-XRPD). The VT-XRPD experiments were performed in symmetrical reflection mode with CuKα radiation (1.54 Å). The scattered intensities were measured with a scintillation counter. The angular range was from 6° to 80° with 0.2° increments, and the measuring time was 3 s/step.

#### 4.4.8. Physical Powder Properties

The density and powder flow properties of spray-dried CH salts were studied by determining and calculating the bulk density, tapped density, Hausner ratio and Carr’s index for the powders. Bulk and tapped densities were determined using an established United States Pharmacopoeia (USP 41, 2018) method [48]. Carr’s index and the Hausner ratio were calculated from the bulk and tapped densities using the following equations: [(ρ_tapped_-ρ_bulk_)/ρ_tapped_] × 100, and ρ_tapped_/ρ_bulk_, respectively [48]. Each sample was measured in triplicate. The experimental data were analysed in accordance with the analysis of variance (ANOVA). When a statistically significant difference (*p* < 0.05) was obtained, a Tukey HSD test was performed.

#### 4.4.9. Chemical Purity Analysis

Ash content, matter insoluble in water, heavy metals, loss on drying and degree of deacetylation (molar) were analysed according to the European Pharmacopoeia (Ph.Eur.) and relevant literature [13,49].

For analysing sulphated ash, 2 g of the sample were incinerated using a muffle (Carbolite, Debyshire, UK) at 750–800 °C for 6 h until the occurrence of total carbonization and the disappearance of white smoke. The final cooling process was carried out in a desiccator and the crucible was weighed. This operation was successfully repeated up to a constant weight. The calculations were expressed on the basis of three replicates. The acceptance limit was set at NMT 1.0%.

The matter insoluble in water was determined by dissolving 1% (*w/v*) chitosan salt in water and then filtering it (filter paper grade 610, Darmstadt, Germany). The filter with the residue was dried at 105 °C until a constant weight was obtained. The calculations were executed on the basis of three replicates. The acceptance limit was set at NMT 0.5%.

For analysing heavy metals, 4 mL of a lead standard solution (10 ppm Pb) were used as a reference solution. The acceptance limit was set at NMT 40 ppm. We used an atomic absorption spectroscopy method described in more detailed in our earlier paper [47]. The calcium, copper, zinc, iron, cadmium, lead, manganese, cobalt and magnesium contents were determined by an atomic absorption spectrophotometer (Avanta P GBC, Braeside, Victoria, Australia) using a hollow cathode lamp of the element. The determination of sodium and potassium was conducted in an emission mode. The least linear squares method was used for calibration.

#### 4.4.10. Loss on Drying

We weighed 1.0 g of the sample (n = 3) on an analytical balance (Sartorius TE 214S, Goettingen, Germany) and placed the sample in an oven (Memmert GmbH, Schwabach, Germany) at 105 °C until a constant weight was obtained. The acceptance limit was set at NMT 10%.

#### 4.4.11. Degree of Deacetylation (DD)

The test solution was prepared by dissolving 0.250 g of CH in purified water (water for analysis) and diluting to 50.0 mL. The solution was stirred vigorously and 1.0 mL of this solution was further diluted to 100.0 mL with purified water. The absorbance of the solution was determined by means of UV spectroscopy at an analytical wavelength of 200–205 nm as the first derivative of the absorbance curve. The quartz cuvettes with a diameter of 1 cm were used in the determination. The pH of the solution was determined.

The reference solutions were prepared at 1.0 μg/mL, 5.0 μg/mL, 15.0 μg/mL and 35.0 μg/mL of N-acetylglucosamine in purified water. The absorbance of each solution was determined at 200–205 nm as the first derivative of the absorption curve. A standard curve was created by plotting the first derivative at 202 nm as a function of the concentration of N-acetylglucosamine and the slope of the least squares linear regression curve was calculated. The standard curve was used to determine the equivalent amount of N-acetylglucosamine for the sample. The DD (molar) was calculated using the following Equation (1):

(1) $$DD(\%)=\frac{100\times M_{1}\times (C_{1}+C_{2})}{(M_{1}\times C_{1})\times \left[(M_{1}+M_{3})\times C_{2}\right]}$$

where C_1_ is the concentration of CH salt in the test solution (µg/mL), C_2_ is the concentration of N-acetylglucosamine in the test solution (µg/mL), M_1_ refers to the relative molecular mass of the N-acetylglucosamine unit in the polymer; M_1_ = 203) and M_3_ refers to the relative molecular mass of the CH salt. M_3_ was calculated from the pH of the solution, taking 6.80 as the pKa value (Equation (2)):

(2) $$M_{3}=f\times M_{2}+\left[\left(1+f)\times (M_{2}+36.5\right)\right]$$

where,

$$f=\frac{p}{1+p}\;,p=10\times (pH-pKa)$$

and M_2_ = 161 (relative molecular mass of deacetylated unit (glucosamine) (C_6_H_11_NO_4_) in polymer).

## 5. Conclusions

Our study confirms that spray drying can be well adapted and scaled up to produce CH salts derived from chitin of lobster origin (*P. argus*) and that the physicochemical properties of the salts are dependent on the organic acid used as a solvent system. The CH acetate and lactate salts can be successfully atomized by optimizing the ratio of inlet air to outlet air temperature and the rotational speed of an atomizer disk. These process parameters are critical for spray drying CH salts in an industrial-scale process and for obtaining a final product of an adequate pharmaceutical excipient quality.

## Figures and Tables

**Table 1:** Summary of statistical analysis.

Variable	*p*-Value
Organic acid used in spray drying (molecular mass, MM)	0.0384
Ratio of inlet /outlet air temperature (difference in temperature, DT)	0.2371
MM:DT	0.7696
Equation of model: Yield = 151.924 − 2.55 MM − 0.14 DT + 0.0023 MM DT r^2^ = 0.83; Adjusted r^2^ = 0.70 Durbin–Watson test = 2.45547 (*p* = 0.9745)	

**Table 2:** Bulk density, tapped density, Hausner ratio and Carr’s index of spray-dried chitosan (CH) acetate and lactate salts. The CH acid salts were spray dried at an inlet/outlet air temperature of 160/100 °C in an industrial-scale process (three parallel batches; mean (standard deviation)).

	CH Acetate			CH Lactate		
Property	B-A001	B-A002	B-A003	B-L001	B-L002	B-L003
Bulk density (g/cm^3^)	0.230 (0.020)	0.250 (0.010)	0.260 (0.020)	0.270 (0.020)	0.260 (0.010)	0.280 (0.000)
Tap density (g/cm^3^)	0.460 (0.020)	0.460 (0.010)	0.470 (0.010)	0.470 (0.010)	0.500 (0.090)	0.460 (0.010)
Hausner ratio	2.00	1.84	1.81	1.74	1.92	1.64
Carr’s index	50.0	45.6	44.7	40.4	48.0	39.1

**Table 3:** Chemical purity of spray-dried chitosan (CH) acetate and lactate salts. The CH acid salts were spray dried at an inlet/outlet air temperature of 160/100 °C in an industrial-scale process (three parallel batches: mean (standard deviation).

Test	CH Acetate			CH Lactate		
	B-A001	B-A002	B-A003	B-L001	B-L002	B-L003
Ash content (%)	0.40 (0.00)	0.39 (0.00)	0.42 (0.00)	0.50 (0.00)	0.50 (0.00)	0.42 (0.00)
Matter insoluble in water (%)	0.35 (0.00)	0.38 (0.00)	0.40 (0.01)	0.30 (0.00)	0.33 (0.00)	0.35 (0.00)
Heavy metals (ppm)	<0.5	<0.5	<0.5	<0.5	<0.5	<0.5
Loss on drying (%)	4.78 (0.03)	4.81 (0.01)	4.84 (0.01)	3.20 (0.01)	3.18 (0.03)	3.21 (0.01)
Degree of deacetylation (molar) (%)	57.36 (0.02)	57.69 (0.01)	57.72 (0.02)	53.82 (0.02)	53.61 (0.01)	53.50 (0.01)

**Table 4:** Matrix of the experimental design.

No.	Run	X_1_	X_2_	Molecular Mass (MM) of Organic Acid (X_1_)	Ratio (Difference) of Inlet/Outlet Temperature, DT (°C) (X_2_)
1	7	−1	+1	60.05	160/100 (60)
2	3	+1	+1	90.08	160/100 (60)
3	1	−1	−1	60.05	140/90 (50)
4	2	+1	−1	90.08	140/90 (50)
5	6	−1	+1	60.05	160/100 (60)
6	4	+1	+1	90.08	160/100 (60)
7	5	−1	−1	60.05	140/90 (50)
8	8	+1	−1	90.08	140/90 (50)

## References

[1] Shahidi F., Arachchi J.K.V., Jeon Y. (1999). Food applications of chitin and chitosans. Trends Food Sci. Technol..

[2] Koide S.S. (1998). Chitin-chitosan: Properties, benefits and risks. Nutr. Res..

[3] Kumar M.N.V.R. (2000). A review of chitin and chitosan applications. React. Funct. Polym..

[4] Muzzarelli R.A.A., Baldassare V., Conti F., Ferrara P., Biagini G., Gazzanelli G., Vasi V. (1988). Biological activity of chitosan: Ultrastructure study. Biomaterials.

[5] Perinelli D.R., Fagioli L., Campana R., Lam J.K.W., Baffone W., Palmieri G.F., Casettari L., Bonacucina G. (2018). Chitosan-based nanosystems and their exploited antimicrobial activity. Eur. J. Pharm. Sci..

[6] Bernkop-Schnürch A., Dünnhaupt S. (2012). Chitosan-based drug delivery systems. Eur. J. Pharm. Biopharm..

[7] Delattre C. (2017). Opinion about advances of chitosan in pharmaceutical field: From past to now. Mod. Appl. Pharm. Pharmacol..

[8] Dutta P.K., Ravikumar M.N.V., Dutta J. (2002). Chitin and chitosan for versatile applications. Polym. Rev..

[9] Ghaz-Jahanian M.A., Abbaspour-Aghdam F., Anarjan N., Berenjian A., Jafarizadeh-Malmiri H. (2015). Application of chitosan-based nanocarriers in tumor-targeted drug delivery. Mol. Biotechnol..

[10] Illum L. (1998). Chitosan and its use as a pharmaceutical excipient. Pharm. Res..

[11] Mohammed M., Syeda Jaweria T.M., Wasan K.M., Wasan E.K. (2017). An overview of chitosan nanoparticles and its application in non-parenteral drug delivery. Pharmaceutics.

[12] Sinha V.R., Singla A.K., Wadhawan S., Kaushik R., Kumria R., Bansal K., Dhawan S. (2004). Chitosan microspheres as a potential carrier for drugs. Int. J. Pharm..

[13] (2011). European Pharmacopoeia.

[14] Rowe R.C., Sheskey P.J., Quinn M.E. (2009). Handbook of Pharmaceutical Excipients.

[15] Bakan J.A., Lachman L., Lieberman H.A., Kaning J.L. (1986). Microencapsulation. The Theory and Practice of Industrial Pharmacy.

[16] Das S.K., Nakka S.R., Rajabalaya R., Mukhopadhyay H.K., Halder T., Palanisamy M., Khanam J., Nanda A. (2011). Microencapsulation techniques and its practice. Int. J. Pharm. Sci. Tech..

[17] Ziaee A., Albadarin A.B., Padrela L., Femmer T., O’Reilly E., Walker G. (2019). Spray drying of pharmaceuticals and biopharmaceuticals: Critical parameters and experimental process optimization approaches. Eur. J. Pharm. Sci..

[18] Illum L., Farraj N.F., Davis S.S. (1994). Chitosan as novel nasal delivery system for peptide drugs. Pharm. Res..

[19] Kissel T., Hilbeert A.K., Koneberg R., Bittner B., Gander B., Merkle H.P., Corradin G. (1997). Microencapsulation of antigens for parenteral vaccine delivery systems. Antigen Delivery Systems: Immunological and Technological Issues.

[20] Ma G. (2014). Microencapsulation of protein drugs for drug delivery: Strategy, preparation, and applications. J. Control. Release.

[21] Desai K.G.H., Park H.J. (2005). Encapsulation of vitamin C in tripolyphosphate crosslinked chitosan microspheres by spray drying. J. Microencapsul..

[22] Fu Y.J., Mi F.L., Wong T.B., Shyu S.S. (2001). Characteristic and controlled release of anticancer drug loaded poly (D, L-lactide) microparticles by spray drying technique. J. Microencapsul..

[23] Mu L., Feng S.S. (2001). Fabrication, characterization and in vitro release of paclitaxel loaded poly (lactic-co-glycolic acid) microspheres prepared by spray drying technique with lipid/ cholesterol emulsifiers. J. Control. Release.

[24] Agnihotri A., Mallikarjuna N., Aminabhavi M. (2004). Recent advances on chitosan-based micro- and nanoparticles in drug delivery. J. Control. Release.

[25] Analava M., Baishakhi D. (2011). Chitosan microspheres in novel drug delivery systems. Indian J. Pharm. Sci..

[26] Aranaz I., Paños I., Peniche C., Heras Á., Acosta N. (2017). Chitosan spray-dried microparticles for controlled delivery of venlafaxine hydrochloride. Molecules.

[27] Di Martino A., Kucharczyk P., Capakova Z., Humpolicek P., Sedlarik V. (2017). Chitosan-based nanocomplexes for simultaneous loading, burst reduction and controlled release of doxorubicin and 5-fluorouracil. Int. J. Biol. Macromol..

[28] Gavini E., Hegge A.B., Rassu G., Sanna V., Testa C., Pirisino G., Karlsen J., Giunchedi P. (2006). Nasal administration of carbamazepine using chitosan microspheres: In vitro/in vivo studies. Int. J. Pharm..

[29] Grenha A., Seijo B., Remuñán-López C. (2005). Microencapsulated chitosan nanoparticles for lung protein delivery. Eur. J. Pharm. Sci..

[30] He P., Davis S.S., Illum L. (1999). Sustained release chitosan microspheres prepared by novel spray drying methods. J. Microencapsul..

[31] Luo M., Peng H., Deng Z., Yin Z., Zhao Q., Xiong H. (2014). Preparation and characterization of genipin-crosslinked chitosan microspheres for the sustained release of salidroside. Int. J. Food Eng..

[32] Pavanetto F., Genta I., Giunchedi P., Conti B., Conte U. (1994). Spray dried albumin microspheres for the intra-articular delivery of dexamethasone. J. Microencapsul..

[33] Shi X.Y., Tan T.W. (2002). Preparation of chitosan/ethylcellulose complex microcapsule and its application in controlled release of Vitamin D2. Biomaterials.

[34] Fernández Cervera M., Heinämäki J., de la Paz N., López O., Maunu S.L., Virtanen T., Hatanpää T., Antikainen O., Nogueira A., Fundora J. (2011). Effects of spray drying on physicochemical properties of chitosan acid salts. AAPS PharmSciTech.

[35] Nunthanid J., Luangtana-anan M., Sriamornsak P., Limmatvapirat S., Puttipipatkhachorn S., Lim L.Y., Khor E. (2004). Characterization of chitosan acetate as a binder for sustained release tablets. J. Control. Release.

[36] Demarger-Andre S., Domard A. (1994). Chitosan carboxylic acid salts in solution and the solid state. Carbohydr. Polym..

[37] Parize A.L., Rozone de Souza T.C., Costa Brighente I.M., de Fávere V.T., Laranjeira M.C., Spinelli A., Longo E. (2008). Microencapsulation of the natural urucum pigment with chitosan by spray drying in different solvents. Afr. J. Biotechnol..

[38] Masters K. (1991). Atomization. Spray Drying Handbook.

[39] Terrizano M. (2002). El Secado de Sólidos en la Industria Química.

[40] Nunthanid J., Huanbutta K., Luangtana-anan M., Sriamornsak P., Limmatvapirat S., Puttipipatkhachorn S. (2008). Development of time-, pH-, and enzyme-controlled colonic drug delivery using spray-dried chitosan acetate and hydroxypropyl methylcellulose. Eur. J. Pharm. Biopharm..

[41] Lorenzo-Lamosa M.L., Remuñán-López C., Vila-Jato J.L., Alonso M.J. (1998). Design of microencapsulated chitosan microspheres for colonic drug delivery. J. Control. Release.

[42] Hirai A., Odani H., Nakajima A. (1991). Determination of degree of deacetylation of chitosan by ^1^H NMR spectroscopy. Polym. Bull..

[43] Prashanth K.V.H., Kittur F.S., Tharanathan R.N. (2002). Solid state of chitosan prepared under different N-acetylating conditions. Carbohydr. Polym..

[44] Muzzarelli R.A.A., Terbojevich M., Muzzarelli C., Francescangeli O. (2002). Chitosans depolymerized with the aid of papain and stabilized as glycosylamines. Carbohydr. Polym..

[45] Poozesh S., Bilgili E. (2019). Scale-up of pharmaceutical spray drying using scale-up rules: A review. Int. J. Pharm..

[46] Cervera M.F., Heinämäki J., Räsänen M., Maunu S.L., Karjalainen M., Nieto Acosta O.M., Iraizoz Colarte A., Yliruusi J. (2004). Solid-state characterization of chitosans derived from lobster chitin. Carbohydr. Polym..

[47] Paz N., Fernández Cervera M., López O., Nogueira A., García C., Pérez D., Tobella J., Montes de Oca Y., Díaz D. (2012). Optimización del proceso de obtención de quitosana derivada de la quitina de langosta. Revista Iberoamericana de Polímeros.

[48] (2018). USP 41—United State Pharmacopeia (USP 41/2018 NF 36).

[49] Sheskey P.J., Cook W.G., Cable C.G., Rowe R.C., Sheskey P.J., Cook W.G., Quinn M.E. (2013). Chitosan. Handbook of Pharmaceutical Excipients.

